# Long-Term Functional Outcomes Following Radiofrequency Microtenotomy for Lateral Epicondylitis of Elbow

**DOI:** 10.7759/cureus.30317

**Published:** 2022-10-15

**Authors:** Shashidharan Viswanathan, Harish Kashyap Shanker

**Affiliations:** 1 Trauma and Orthopaedics, Royal Alexandra Hospital, Paisley, GBR

**Keywords:** coblation, radiofrequency microtenotomy, tendinosis, tennis elbow, lateral epicondylitis elbow

## Abstract

Background

Lateral epicondylitis or ‘Tennis elbow’ is a common cause of elbow pain in the middle-aged group caused by tendinosis of the common extensor origin of the forearm muscles. Though no obvious aetiology is identified in most cases, it could be attributed to repetitive overuse of wrist extensors or supinator muscles. This condition is generally self-limiting but may become persistent in a few cases. Radiofrequency microtenotomy (RFM) is a minimally invasive surgical procedure for recalcitrant lateral epicondylitis of the elbow. This involves targeted coblation of pathological tissue at significantly lower temperatures.

Objectives

The aim of this study was to evaluate the therapeutic efficacy and report long-term results and recurrences in patients treated with RFM.

Methods

We present long-term results with a mean eight-year follow-up in a case series of 19 patients. All patients had a minimum of six months (mean 23.25 months and range: 6-36 months) of conservative management which included steroid injections prior to being offered RFM. This was a retrospective case series of 20 elbows (in 19 patients) who underwent RFM. The majority of patients (65%) were females. The operation was carried out in the dominant arm in 55% of patients.

Results

Results were analysed by comparing pre-operative and post-operative QuickDASH scores (Disabilities of the Arm, Shoulder and Hand Score) obtained at one year and eight years post-operatively. We found an improvement in QuickDASH scores from a mean of 61.7 pre-operatively to 18.9 (p-value < 0.0001) and 8.5 (p-value < 0.0001) at one year and eight years, respectively. The mean pain component of the QuickDASH scores decreased from 4.8 to 2.0 and 1.5, respectively, at one year and eight years (p-value < 0.0001). More than 83% of the patients had excellent to good functional improvement.

Conclusion

RFM is a reliable modality for treating recalcitrant lateral epicondylitis of the elbow with excellent long-term results.

## Introduction

Lateral epicondylitis is a form of tendinosis rather than tendinitis, though these terms are often used interchangeably for clinical diagnosis and are classed as tendinopathies. The difference between tendinitis and tendinosis is histological. Tendinosis is characterised by the absence of inflammatory cells like macrophages, lymphocytes and neutrophils [1]. Tendinosis also has active fibroblasts with vascular hyperplasia unlike tendonitis and therefore are labelled as angioblastic hyperplasia [2].

Multiple repetitive micro traumatic events cause degeneration of cells, and matrix and disrupt the internal structure of the tendon [3]. It is also believed that with age, the cells frequently shift from aerobic to anaerobic energy production increasing the matrix metalloproteinase (MMP) production and leading to an imbalance between tendon destruction and composition, with an increase in matrix degeneration [4]. The tendons of the extensor carpi radialis brevis (ECRB) and anterior third of extensor communis at the elbow are commonly involved [5].

Non-operative interventions include physiotherapy with eccentric exercises [6], epicondylar clasp, anti-inflammatories, steroid injections, and infiltration of platelet-rich plasma (PRP) [7]. Other methods described include extracorporeal shock wave therapy (ESWT), percutaneous radiofrequency ablation and topical nitrates [8].

Surgical intervention is recommended only after a trial of non-operative management with persisting symptoms affecting the quality of life (QOL). The radiofrequency microtenotomy (RFM) device works on the principle that it creates a precise and small plasma bubble when micro voltages are applied [9]. This leads to coblation which dissolves soft tissues by breaking molecular bonds at significantly lower temperatures than the traditional radiofrequency techniques [10].

## Materials and methods

This was a retrospective case series (level of evidence [LoE IV]) study that involved the recruitment of 24 patients over a two-year period from June 2012 to June 2014. Lateral epicondylitis was diagnosed clinically. Patients had tenderness over common extensor origin at the elbow and had pain on resisted wrist extension. This study was exempt from the National health service (NHS) Research Ethics Committee review. IRAS (Integrated Research Application System) Project ID: 321176.

Inclusion criteria were persistent symptoms of lateral epicondylitis after six months of conservative management which included administration of analgesics, physiotherapy, epicondylar clasp and local steroid injections. Three patients with simultaneous medial epicondylitis in the ipsilateral elbow were excluded. Two patients were lost for follow-up. None of the patients included in the study suffered from seropositive (rheumatoid arthritis, lupus, etc.,) or seronegative (psoriasis, ankolysing spondylitis, reactive arthritis) or crystal-induced (gout, pseudogout) inflammatory arthropathy. QuickDASH scores were obtained from these patients preoperatively.

The mean duration of non-operative management was 23 months (range: 6-36 months). The majority (79%) of patients had three or more steroid injections pre-operatively. The final analysis included 19 patients who were all followed up for a mean of eight years and one patient had the procedure bilaterally at separate sittings (three months apart). The mean age at RFM surgery was 48.8 years (range: 42-69 years).

RFM was done using the TOPAZ® EZ MicroDebrider (ArthroCare Corp., Austin - TX; USA). This is a single-use microdebrider that has integrated finger switches for activation in the tissue. The tip diameter was one millimetre. Saline was delivered from irrigation tubing on the wand to the ablation site through fenestrations in the four centimetres working length of the wand, just above the tip. This was a minimally invasive procedure that involves coblating the area of tendinopathy. The procedure involved the application of radiofrequency energy and precise removal of tissue with minimal damage.

All procedures were performed by or under the direct supervision of the senior author. The painful tender area was marked by palpation pre-operatively before the administration of anaesthesia. General anaesthesia and an arm tourniquet were used with the patient in a supine position with the arm placed on a hand table. A skin incision of approximately 3 to 4 cm in length was made centred over the marked tender area with dissection carried out to expose the common extensor origin.

TOPAZ coblator wand was plugged into the controller, which was set up by connecting to a power source. A bag of isotonic saline was connected to the irrigation tubing on the wand. The flow was set at two to three drops per second.

RFM applications were delivered to the symptomatic area on the tendon at 2 to 3 millimetres intervals at varying depths in millimetres. The wand was held perpendicular to the surface during fenestrations and activated for 0.5 seconds with light pressure. This created a grid of fenestrations over an area covering two square centimetres. The wound was washed with isotonic saline and the skin was closed with interrupted non-absorbable sutures. Local anaesthetic was infiltrated for post-operative pain management. Sterile dressings were applied.

The use of non-steroidal anti-inflammatory agents was avoided to prevent the blockade of the inflammatory response which follows the procedure. Patients were reviewed by the physiotherapist on the ward prior to discharge and were given follow-up appointments for physiotherapy. Patients were encouraged to start full range of motion (ROM) exercises and avoid heavy lifting post-operatively for six weeks. The skin sutures were removed by nurse practitioners at two weeks and patients were followed up at three months in the shoulder and elbow clinic of the senior author. Patients were given an open return appointment if asymptomatic at any point. QuickDASH scores were obtained at one year and a mean of eight years postoperatively. The mean duration of follow-up was 8 years (range: 7-9.33 years).

## Results

The final analysis included 19 patients who were all followed up for a mean of eight years and one patient had the procedure bilaterally at separate sittings (three months apart). The mean age at RFM surgery was 48.8 years (range: 42-69 years). This included seven males and 12 females. The operation was carried out in the dominant arm in 11 (i.e., 55%) patients.

**Figure 1:**
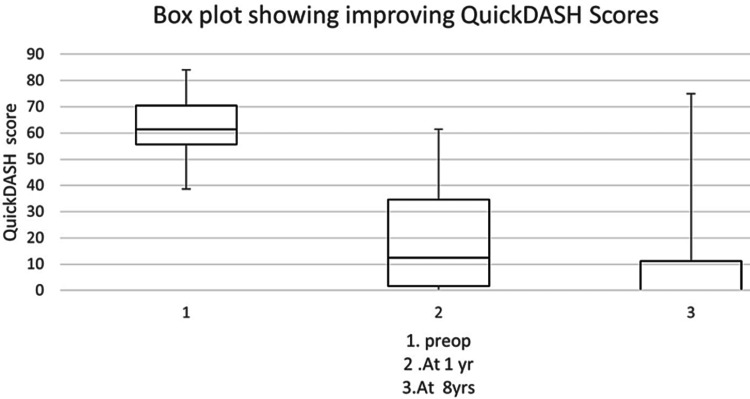
Boxplot showing improving QuickDASH scores

The mean QuickDASH scores decreased from 61.7 (range: 38.6 to 84.1) to 18.9 (range: 0 to 61.4) at one year (Table 1, Figure 1) (T-test showed a p-value < 0.0001). QuickDASH scores decreased further to 8.5 (range: 0 to 75) at eight years (T-test showed a p-value < 0.0001).

**Table 1:** QuickDASH score

	Preoperative	One year Post-op (n=20)	Eight year post-op (n=18)	P-value (Paired T test)
QuickDASH	61.7	18.9	8.5	<0.0001
Pain component of QuickDash score (1-5)	4.8 / 5	2.0 / 5	1.5 / 5	<0.0001

**Figure 2:**
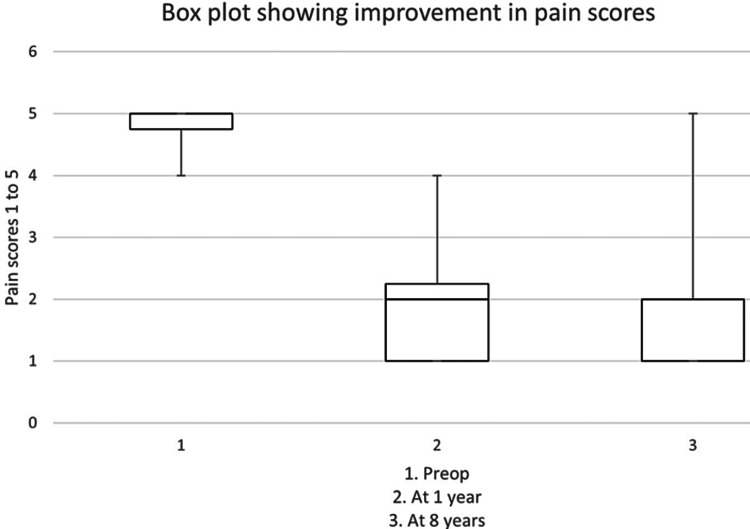
Box plot showing improvement in pain scores

The mean pain component of the QuickDASH scores decreased from 4.8 to 2.0 (out of 5.0) at one year (T-test showed a p-value < 0.0001) (Table 1, Figure 2). The pain component of the QuickDASH scores decreased further to 1.5 (out of 5.0) at eight years (T-test showed a p-value < 0.0001).

More than 83% of the patients had excellent to good functional improvement (QuickDASH scores improved by 75% or more) at eight years (Table 2).

**Table 2:** Functional improvement

Percentage of decrease in QuickDASH Score	Number of patients	Percentage of patients
Excellent (>87% decrease)	13	72.22%
Good (75% - 90% decrease)	2	11.11%
Fair (50% to 74% decrease)	2	11.11%
Poor (50% decrease)	1	5.55%

One patient developed a superficial wound infection which settled with debridement under local anaesthesia and oral antibiotics within a couple of weeks. At one year follow-up, two patients had persistent pain of which one chose to have the open release of ECRB. At a further follow-up of eight years, another patient underwent open release for recurrence (after being symptom-less and pain-free for two years post-RFM). Both patients had symptomatic relief post-open release.

Despite the relatively small number of subjects in this group, a significant improvement in pain, and return to work was noted in the majority of patients. A large number (89.47%) of patients were happy with the results and were happy to recommend it to others or have the same procedure performed on the opposite side (if they were to become symptomatic).

## Discussion

RFM was successful in 83.33% of patients who had good or excellent results at eight years post-surgery. The pain was reduced by 58.33% post-operatively at one year and 68.75% at eight years. Revision surgery in the form of open release was undertaken in only two patients at long-term follow-up of eight years (partial relief of symptoms in one of them and recurrence at two years in the other patient). Other complications included a superficial infection (one patient) that resolved with local debridement and oral antibiotics.

Lateral epicondylitis is a self-limiting condition with a natural history of 10-18 months for subsidence of symptoms [11]. In a small minority of patients, the symptoms may persist warranting some form of intervention despite steroid injections and other non-operative treatments. Ultrasound and MRI have been shown to have a poor correlation with patients’ symptoms [12].

Various operative procedures in the form of open vs. arthroscopic procedures have been carried out over the years. Tasto et al. reported microtenotomy using a radiofrequency probe is a safe and effective procedure for elbow epicondylitis in 91% of patients with successful outcomes at nine years [13]. Koh et al. reported a case series of 20 patients who underwent ultrasonic microresection for recalcitrant lateral elbow tendinopathy with 95% good results at one year [14]. Seitz et al. undertook a prospective study of 40 patients treated with radiofrequency coblation and localized arthroscopic synovectomy [15]. A synovitic ‘plica’ unfolding into the radio-capitellar joint was seen in 82.5% patients which caused erosive changes in the postero-lateral radial head. They reported an average drop of 7.7 points (on a visual analog scale -VAS of 0-10) six months after the operation. Lee et al. undertook a randomised controlled trial of 55 patients to compare the clinical effects of radiofrequency-based microtenotomy vs. arthroscopic release of the ECRB tendon in patients with recalcitrant lateral epicondylitis [16]. They concluded that RFM provided clinical outcomes comparable with those achieved with the arthroscopic release of ECRB tendon during the recovery phase. Hamlin et al. in a randomised control trial of 41 patients compared the outcomes in RFM and Open release in refractory cases of lateral epicondylitis [17]. They concluded that these patients treated with both methods had significant improvement in symptoms and similar outcomes. No statistical difference was found in Numerical Rating Scale (NRS) pain score, grip strength and Disabilities of the Arm, Shoulder and Hand (DASH) scores post-operatively at one year. Our study showed similar functional results and reduction in pain in comparison to other studies for this treatment (Table 3).

**Table 3:** Comparison with other studies

Study	Number operated	Mean follow up duration	Improvement in pain score	Mean Improvement in Functional scores in points out of 100	Patients with persistent pain
Tasto et al. [13]	69	2.5 yr	81%	Not done	6
Koh et al. [14]	20	1 yr	90%	DASH score – 19 points	1
Seitz et al. [15]	40	2.8 yr	85%	MEPS^*^ score - 40 points	2
Lee et al. [16]	22	2 yr	72%	MEPS^*^ score - 41.3 points	1
Hamlin et al. [17]	23	1 yr	71%	DASH score - 39.5 points	2
This study	20	At 1 yr	58%	QuickDASH score – 42.8 points	2
This study	20	At 8 yr	69%	QuickDASH score – 51.7 points	3
MEPS^*^ score - Mayo Elbow Performance score					

The strength of our work lies in the significant length of follow up we have been able to achieve, albeit with a small cohort. We also had strict inclusion criteria, with all patients having failed a significant period of nonoperative treatment prior to surgery.

However, our study is not without limitations. We had a relatively small study cohort of 19 patients. However, our study showed a significant improvement in QuickDASH scores considering, MDC95 (minimal detectable change at the 95% confidence level) for QuickDASH scoring ranged from 16 to 20 QuickDASH points (with a mean of 18) [18]. Further prospective studies with larger numbers would be needed.

## Conclusions

We conclude that RFM is a minimally invasive and reliable modality of treating recalcitrant lateral epicondylitis of the elbow that yielded good to excellent results in long term without any permanent sequelae or complications after failed conservative management. Further prospective studies involving a larger number of patients are needed to evaluate the therapeutic efficacy of this treatment.
